# Impact of immunohistochemistry-based molecular subtype on chemosensitivity and survival in Hispanic breast cancer patients following neoadjuvant chemotherapy

**DOI:** 10.3332/ecancer.2015.562

**Published:** 2015-08-06

**Authors:** Rodolfo Gómez, Carlos Andrés Ossa, María Elvira Montoya, Carolina Echeverri, Gonzalo Ángel, Johana Ascuntar, Mauricio Borrero, Mónica Gil, Sabrina Herrera, Eduardo Gutiérrez, Fernando Herazo, Alejo Jiménez, Jorge Madrid, Pedro Alejandro Reyes, Lina Zuluaga, Héctor García

**Affiliations:** 1 Instituto de Cancerología-Clínica Las Américas, Medellín 05001000, Colombia; 2 School of Medicine, Universidad de Antioquia, Medellín, Colombia; 3 Cancer Research Group, Instituto de Cancerología-Clínica Las Américas, Medellín 05001000, Colombia; 4 School of Medicine, Clinical Epidemiology Academic Group (GRAEPIC), Universidad de Antioquia, Medellín, Colombia

**Keywords:** breast cancer, Colombia, Hispanics, intrinsic subtypes, molecular subtypes, neoadjuvant therapy, pathological complete response

## Abstract

**Background:**

Neoadjuvant chemotherapy (NAC) is the standard treatment for patients with locally advanced breast cancer, showing improvement in disease-free survival (DFS) and overall survival (OS) rates in patients achieving pathological complete response (pCR). The relationship between immunohistochemistry-based molecular subtyping (IMS), chemo sensitivity and survival is currently a matter of interest. We explore this relationship in a Hispanic cohort of breast cancer patients treated with NAC.

**Methods:**

A retrospective survival analysis was performed on Colombian females with breast cancer treated at Instituto de Cancerología-Clinica Las Américas between January 2009 and December 2011. Patients were classified according to immunohistochemistry-based subtyping into the following five groups: Luminal A, Luminal B, Luminal B/HER 2+, HER2-enriched, and triple-negative breast cancer. Demographic characteristics, recurrence pattern, and survival rate were reviewed by bivariate and multivariate analysis.

**Results:**

A total of 328 patients fulfilled the study’s inclusion parameters and the distribution of subtypes were as follows: Luminal A: 73 (22.3%), Luminal B/HER2−: 110 (33.5%), Luminal B/HER2+: 75 (22.9%), HER2-enriched: 30 (9.1%), and triple-negative: 40 (12.2%). The median follow-up was 41 months (interquartile range: 31–52). Pathological response to NAC was as follows: complete pathological response (pCR) in 28 (8.5%) patients, partial 247 (75.3%); stable disease 47 (14.3%), and progression 6 (1.8%) patients. The presence of pCR had a significant DFS and OS in the entire group (*p* = 0.01) but subtypes had different DFS in Luminal B (*p* = 0.01) and triple negative (*p* = 0.02) and also OS in Luminal B (*p* = 0.01) and triple negative (*p* = 0.01).

**Conclusions:**

pCR is associated with an improved overall survival and disease-free survival rates in this group of Hispanics patients. Advanced stages, Luminal B subtypes, triple-negative tumours and non-pCR showed lower DFS.

## Introduction

Neodjuvant chemotherapy (NAC) has proven either to decrease tumour size in cancer patients not suitable for primary surgery or to increase the possibility of conservative surgery when it had previously been inadvisable. Currently, it must be considered an evaluative tool in designing a therapeutic strategy or biological factors that may help to determine the prognosis and treatment alternatives in a given patients.

NAC has shown as significantly improved disease-free survival (DFS) and overall survival (OS) rates as adjuvant therapy in patients with breast cancer [1]; moreover, it has become the standard procedure when handling non-surgical tumours and inflammatory carcinomas [2]. It increases the proportion rate of conservative surgeries in patients with locally advanced breast cancer tumours [3] and decreases resection extension even in patients scheduled for conservative surgery [4].

In the last few years, breast cancer intrinsic molecular subtypes have been evaluated by gene expression array data, a technology not widely available worldwide. Immunohistochemistry-based molecular subtype with ER, PR, Ki67, and HER2 Neu has shown good correlation with gene expression assays to identify intrinsic subtypes [5].

Previous reports state that not all patients have an equal response to NAC and that this response may vary depending on biological characteristics of the tumour and their immunohistochemistry-based molecular subtype, In basal-like tumours achieving a complete pathological response (pCR) rate in 45% (95%CI: 24–68), HER2+ in 45% (95%CI: 23–68), and Luminal A in 6% (95%CI:1–21) [6].

Some publications suggest that pCR could be a surrogate marker for OS, particularly in the triple-negative and HER2 subgroups, with HER2+/hormone receptor (HR)-negative presenting a higher OS rate than HER2+/HR+ patients [7]. Although pCR has proven to be consistently associated with an excellent survival rate, it is not the only prognostic factor, as in cases of less aggressive tumours like Luminal A.

Achieving pCR at the time of surgery has been associated with a favourable prognosis [8, 9]. Not achieving pCR has shown worse results in triple-negative and HER2+ tumours, although this prognostic correlation has not been observed in HR+ tumours [9, 10].

This study aims to determine the pathological response and its correlation with breast cancer immunohistochemistry-based molecular subtype as well as its correlation with DFS and OS rates in patients who have undergone NAC at Instituto de Cancerología- Clínica Las Américas (IDC), a comprehensive cancer centre in Colombia.

## Patients and Methods

A retrospective survival study was performed in women older than 18 years of age treated with NAC at the IDC between January 2009 and December 2011.

**Figure 1.**
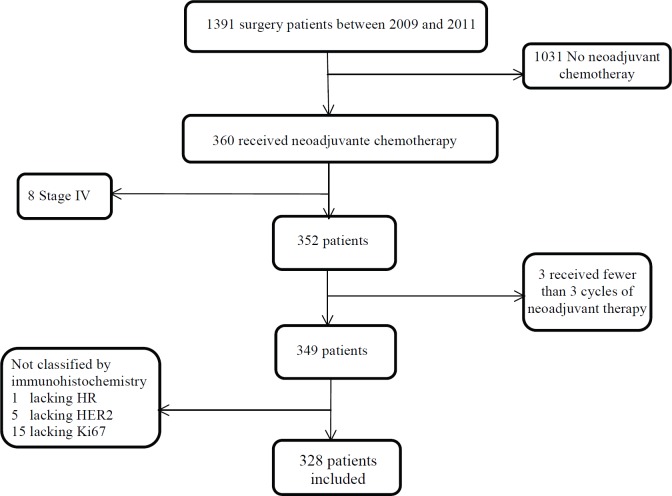
Study profile.

The study included patients with histological diagnosis of breast cancer, Stages II and III, who received NAC, breast surgery and had a histopathological assay in our centre (Figure 1). Neither patients with Stage IV at the time of diagnosis nor patients receiving fewer than three NAC cycles were included. The same was for patients without complete IHC information available.

All demographic, clinical, and follow-up variables were obtained from the IDC patient’s registry, medical records, and surgical pathology reports. Pathology reports follow the College of American Pathologists (CAP) checklists. The ER, PR, and HER2 Neu were evaluated following the published CAP guideline recommendations [11, 12]. Antibody clones were used: ER clone SP1 (Thermo scientific, USA), PR clone 16 (Leica Biosystems, Germany), Monoclonal Mouse Anti-Human Ki-67 Antigen (Dako, Denmark), and HER-2/neu (4B5) Rabbit Monoclonal Primary Antibody (Ventana, USA) was used in the majority of cases. The Ki-67 was performed in some cases when it was not carried out in initial pathology study, using the MIB1 antibody (Dako, Glostrup, Denmark). Ki-67 cut-off to separate low and high risk was 14% as proposed by Consensus [13].

Patient’s vital status was traced through phone calls. IDC´s IRB approved the research project.

Hormone receptor status (ER and PR) is considered positive if IHC staining is ≥1%. HER2 is positive in tumours showing IHC +++ or IHC ++ by positive FISH (≥ 2.4).

Immunohistochemistry-based molecular subtypes definition:

‘Luminal A’: ER+ and/or PR+, HER2−, Ki-67 < 14%
‘Luminal B’: ER+ and/or PR+, HER2−, Ki-67 ≥ 14%
‘Luminal B/HER 2+’: ER+ and/or PR+, HER2+
‘HER2-enriched’: ER− and PR−, HER2+
‘Triple negative’: ER−, PR−, HER2−

The chemotherapy schema utilised were znthracyclines and taxanes, anthracyclines alone, and taxanes only. Patients who had contraindicated the use of anthracyclines was included in the taxane scheme (docetaxel–cyclophosphamide) or treated with cyclophosphamide, methotrexate, and fluorouracil (CMF).

As described in a recent paper, a pCR was defined as ypT0 ypN0 (the absence of invasive cancer and *in situ* cancer in the breast and axillary nodes) [14].

## Statistical Analysis

Bivariate analysis by intrinsic subtypes was performed. The chi-square or Fisher’s exact test were used for the categorical variables, and the *t*-test or one-way analysis of variance (ANOVA) was used for numeric variables. Survival rate was calculated up until the date of relapse, death, or last control check. Kaplan–Meier curves were assessed according to pCR, subtypes and clinical variables that were compared with a Breslow test. In the multivariate analysis, all variables with clinical or statistical significance (*p* < 0.25) in bivariate analysis were included. All variables were categorised for multivariable analysis. Adjusting for potential confounders was performed with Cox regression analysis. The statistical significance for the variables included in the multivariate model was set at a level of statistical significance of *p* < 0.05. A two-tailed *p* value was established at > 0.05. SPSS software (version 20.0; SPSS, Chicago, IL) was used in the statistical analysis.

## Results

Table 1 shows the population´s clinical characteristics. A total of 328 patients within 24–81 years of age fulfilled the inclusion criteria. The mean age was of 52.9 years (standard deviation: 11.3), and 83 (25.3%) patients were younger than 45 years of age.

Sixty-five (19.8%) patients were Stage II and 263 (80.2%) Stage III; tumour size was ≥ 30 mm in 93.8% of patients. The chemotherapy schema used was anthracyclines and taxanes in 272 (82.9%), anthracycline alone in 46 (14.1%), taxanes only in 6 (1.8%) and CMF in the remaining 4 (1.2%) patients. Out of 105 (32%) patients with HER2-positive cases 87 (82.9%) received trastuzumab in the neoadjuvant setting.

After neoadjuvant chemotherapy, 226 (68.9%) patients had mastectomy, 37 (11.3%) of them with early reconstruction, and breast-conserving surgery in 102 (31.1%). According to our institutional protocol at that time, axillar dissection was performed directly in 307 (93.6%) cases, and sentinel lymph node post-NAC was performed in only 28 (8.5%) patients.

Involved lymph nodes were found in 187 (57.7%) cases: 88 had 1–3, 60 from 4–9, and in 39, more than nine were involved.

Immunohistochemistry-based molecular subtype distribution was as follows: ‘Luminal A’: 73 (22.3%); ‘Luminal B’: 110 (33.5%); ‘Luminal B/HER2+’: 75 (22.9%); ‘HER2-enriched’: 30 (9.1%); and ‘triple negative’: 40 (12.2%). Table 2 shows the clinical characteristics according to intrinsic subtype.

Median time elapsed between histopathological diagnosis and NAC was 56 days (IQR: 34–103), median time of duration of NAC was 175 days (IQR: 144–193). 271 (82.6%) patients received the whole-planned chemotherapy schema. Of the remaining 57 cases, 15 had drug intolerance, 10 disease progression, 13 poor drug response, six due insurance coverage issues, six because of patients desire, and seven for different comorbidities.

Overall frequency of pCR was 8.5% (*n* = 28), partial responses in 75.3% (*n* = 247), the disease remained stable in 14.3% (*n* = 47), and there was progression in 1.8% (*n* = 6) patients. The rates of pCR differed (*p* < 0.01) among subtypes: 27.5% of ‘Triple negative’, 13.3% of ‘HER2-enriched’, 13.5% of ‘Luminal B/HER2+’, 2.7% of ‘Luminal B’, and none in ‘Luminal A’ (Table 3).

In 328 patients, we evaluate the Ki-67 ≤ 14. Among the 140 patients with Ki-67 ≤ 14, 20/140 patients (14.3%) had pCR, versus 8/188 patients (8.5%) had pCR with Ki-67 >14 (*p* < 0.05). despite finding statistical significance. In multivariate analysis, no association was found in DFS and OS for ki67.

About 225 of our patients (68.6%) receive hormonotherapy as a adjuvant treatment, tamoxifen (202) was the upfront medication in this group of patients in the majority of the cases 89.9%.

Median follow-up was 41 months (IQR: 31–52). Alive without disease evidence 213 (64.9%), alive with active disease 26 (7.9%), 78 (23.8%) had died, and 11 (3.3%) were missing from the follow-up.

**Figure 2.**
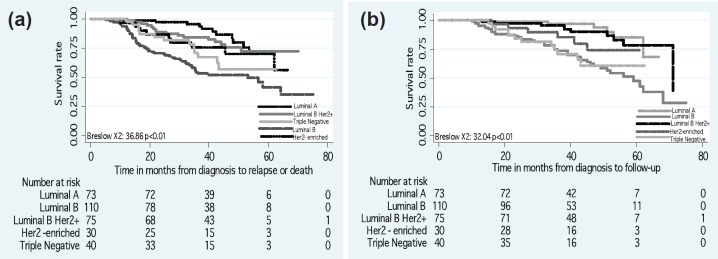
(a) Disease-free survival and (b) overall survival by intrinsic subtypes.

**Figure 3.**
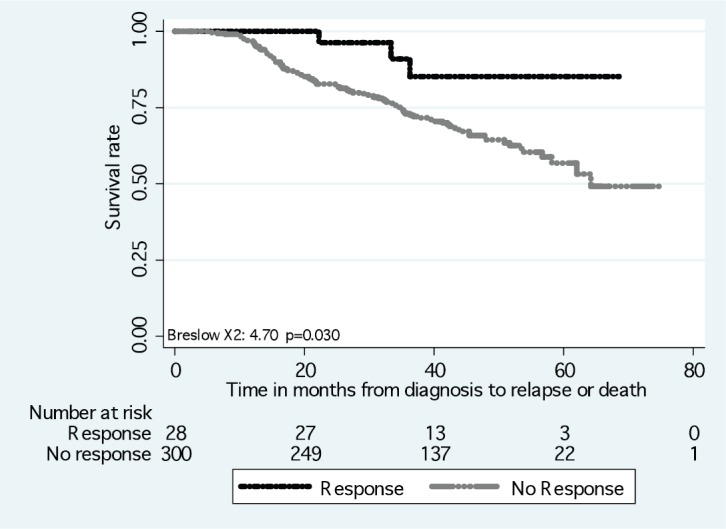
Disease-free survival in response to neoadjuvant chemotherapy.

The DFS and OS rates differed according to the intrinsic subtype (*p* < 0.01) (Figure 2). Differences in the DFS rate were found between women who achieved pCR versus non-pCR (*p* = 0.03) (Figure 3), but not OS rate (*p* > 0.05).

The multivariable models are depicted in Table 4. After adjusting for confounders, the OS was poor in patients did not achieve pCR to NAC (HR: 5.43; 95%CI: 1.26–23.3); ‘Luminal B subtype’ (HR: 5.12; 95%CI: 2.18–12.05); ‘triple-negative’ subtype (HR: 7.41; 95%CI: 2.73–20.14), and clinical Stage III (HR: 3.43; 95%CI: 1.38–8.54). The variables associated with worse DFS were not achieve pCR to NAC (HR: 3.96; 95%CI 1.21–12.9); ‘Luminal B’ subtype (HR: 3.19; 95%CI: 1.66–6.14); ‘triple-negative’ subtype (HR: 2.62; 95%CI: 1.14–6.00) and histological grade 3 (HR: 1.66; 95%CI: 1.09–2.54).

## Discussion

In the analysis of the results of our study, some important characteristics from the patients treated with NAC in our centre were considered. First, 80.2%, in patients had locally advanced tumours (Stage III), implying a larger tumour load and lower survival expectancy. This could also explain why the most common surgeries for these patients were mastectomy (68.9%).

The immunohistochemistry-based molecular subtype distribution of our patients, both in this neoadjuvant group and in our database [15] show differences from previously published data of developed countries [10, 16–17] and even with other Latin American series such as Mexico that report only 57% of positive hormone receptors (HR), Brazil 55% and Costa Rica 49% [18, 19]. We have a higher frequency of patients with positive HR 78.7% (55.8% HER2+ and 22.9% HER2−) who were often in Stage IIIB (63% ‘Luminal A’, 55.5% ‘Luminal B’ and 56% ‘Luminal B/HER2+’). We also have a lower frequency of the ‘triple negative’ (12.2%) and ‘Her2-enriched’ (9.1%) subtypes.

Our overall frequency of pCR was low, that is 8.5%, and is inferior to the values reported by other groups (13%–15%) [10, 20, 21], but their patients exhibited earlier stage disease than ours (71% T1–T2, 95% N0–N1). In those patients treated with NAC, the results seem to suggest that there are differences in chemotherapy response according to intrinsic subtype. In our study, the pCR frequency was 27.5% in the ‘triple-negative’ group, 13.3% in ‘Her2-enriched’ treated with chemotherapy and trastuzumab and 13.3% in ‘Luminal B/HER2+’ using the same treatment. In comparison, patients with the ‘Luminal A’ subtype had 0 pCR, and ‘Luminal B’ 2.7%. These differences reached a significant value compared with the Her2+ groups in which we use trastuzumab. Likewise, the triple-negative groups also showed significant differences compared with the positive hormone receptors group.

Other papers have reported improved long-term outcomes in patients with pCR [6, 8, 10, 20–22]. In this report, pCR was associated with a significant increase in DFS and OS rates. The German pooled analysis and CTNeoBC pooled analysis also shown these association [10, 16]. The ‘Luminal B’ breast cancer has been recognised as having worst prognosis, these characteristics were similar in our cohort of patients, but superior to ‘HER2- enriched’ and ‘basal-like tumours’ [23].

The association of pCR with an improved DFS rate is observed in the literature [10, 20, 21, 23, 25] and occurs in our study as well as the association with OS rate reported by Cortazar *et al* [21].

The immunohistochemistry-based molecular subtype distribution in our series shows important differences with the German series [10]: ‘Luminal A’ was 39% in theirs versus 22.3% in ours, ‘Luminal B’ 8.5% versus 33.5%, ‘Luminal B/HER2+’ 17.9% versus 22.9%, ‘HER2-enriched’ 12.8% versus 9.1%, and ‘triple negative’ 21.7% theirs versus 12.2% ours. It is evident, therefore, that we are addressing populations whose disease possesses different characteristics and in which we can find different results.

## Conclusion

In conclusion, it is evident that for our patients, pCR is associated with an improved DFS and OS rate. In our cohort, achievement of pCR was more frequent in ‘triple negative’ and ‘HER2-enriched’ patients, as has been previously shown in other studies. We believe that the low pCR rate in our trial was due to the advanced cancer stages in the majority of our population (80.2% were Stage III). This needs to be confirmed in prospective studies. We do not have an explanation for the high frequency of HR+ tumours in our population. It could be ethnic and deserves further investigation.

## Conflicts of Interests

None declared.

## Funding

Instituto de Cancerología- IDC, Comprehensive Cancer Center in Colombia.

## Authors´ Contributions

The concept and design of the study was contributed by Rodolfo Gómez, Carlos Andrés Ossa, Héctor García. Rodolfo Gómez, Carlos Andrés Ossa, Fernando Herazo, Carolina Echeverri, Alejo Jiménez, Jorge Madrid, Mónica Gil, Sabrina Herrera María Elvira Montoya, Gonzalo Ángel, Mauricio Borrero, Pedro Reyes, and Eduardo Gutiérrez were involved in material supply and patient referral. Data collection and processing was done by Lina Zuluaga, Johana Ascuntar. Data analysis and interpretation were performed by Rodolfo Gómez, Carlos Andrés Ossa, Héctor García. Rodolfo Gómez, Carlos Andrés Ossa, Fernando Herazo, and Héctor García were involved in the final manuscript approval.

## Figures and Tables

**Table 1.** Clinical characteristics of population.

Characteristics	
**Mean age (sd)**	52.9 (11.3)
**Age groups**	–0.6
< 45 years	83 (25.3 %)
45 - 54 years	103 (31.4 %)
≥ 55 years	142 (43.3 %)
**Clinical stage**	
IIA	25 (7.6 %)
IIB	40 (12.2 %)
IIIA	68 (20.8 %)
IIIB	183 (55.7 %)
IIIC	12 (3.7 %)
**Histological variety**	
Ductal	300 (91.5%)
Lobular	17 (5.2 %)
Other	11 (3.3 %)
**Histological grade**	
1	46 (14.0 %)
2	122 (37.2 %)
3	160 (48.8 %)
**Tumour size (mm)**	
< 30	19 (6.2 %)
≥ 30	288 (93.8 %)
**Type of surgery**	
Quadrantectomy	102 (31.1 %)
Mastectomy	226 (68.9 %)
Reconstruction	37 (16.4 %)
**Sentinel lymph node**	28 (8.5 %)
**Axillary clearance**	307 (93.6 %)
**Positive lymph nodes at clearance**	
Negative	120 (39.1 %)
1 to 3	88 (28.7 %)
4 to 9	60 (19.5 %)
≥ 10	39 (12.7 %)
**Hormone receptor status**	
Estrogen receptor positive	252 (76.8 %)
Progesterone receptor positive	197 (60.1 %)
**HER2/neupositive**	105 (32.0 %)
**KI-67 score**	
≤ 14	140 (42.7 %)
> 14	188 (57.3 %)
**Neoadjuvant chemotherapy regimen**	
Anthracycline-based only	46 (14.0 % )
Anthracycline and taxane based	272 (82.9 %)
Taxane-based only	6 (1.8 %)
CFM^*^	4 (1.2 %)
**HER2/neu–Trastuzumab**	
HER2/neu–Yes Trastuzumab	89 (84.8 %)
HER2/neu–No Trastuzumab	16 (15.2 %)
**Metastasis and/or relapse**	
Local	9 (11.8 %)
Metastasis	67 (88.2 %)

* CFM: cyclophosphamide, methotrexate, and fluorouracil

**Table 2.** Response to neoadjuvant chemotherapy by molecular subtypes.

	Luminal A 73 (22.3) # (%)	Luminal B 110 (33.5) # (%)	Luminal B HER2 + 75 (22.9) # (%)	HER2-enriched 30 (9.1) # (%)	Triple negative 40 (12.2) # (%)	Total 328 # (%)	*p* value
**Response to NAC**							< 0.01
NAC progression	0 (0.0)	3 (2.7)	0 (0.0)	2 (6.7)	1 (2.5)	6 (1.8)	
Stable disease	6 (8.2)	24 (21.8)	10 (13.3)	3 (10.0)	4 (10.0)	47 (14.3)	
Partial response	67 (91.8)	80 (72.7)	55 (73.3)	21 (70.0)	24 (60.0)	247 (75.3)	
Complete response (pCR)	0 (0.0)	3 (2.7)	10 (13.3)	4 (13.3)	11 (27.5)	28 (8.5)	

*p*-value for the comparison of pCR versus non-pCR between subtypes

**Table 3.** Clinical characteristics of intrinsic subtypes.

Characteristics	Luminal A 73 (22.3%) # (%)	Luminal B 110 (33.5%) # (%)	Luminal B/HER2+ 75 (22.9%) # (%)	HER2-enriched 30 (9.1%) # (%)	Triple negative 40 (12.2%) # (%)	*p*-value
**Mean age (de)**	51.9 (9.6)	54.1 (11.4)	51.7 (11.8)	51.4 (11.2)	54.6 (13.0)	0.40
**Age groups**						
< 45 years	16 (21.9)	26 (23.6)	22 (29.3)	10 (33.3)	9 (22.5)	0.14
45 - 54 years	31 (42.5)	28 (25.5)	26 (34.7)	9 (30.0)	9 (22.5)	
≥ 55 years	26 (35.6)	56 (50.9)	27 (36.0)	11 (36.7)	22 (55.0)	
Clinical stage^*^						
IIA	8 (10.9)	6 (5.5)	5 (6.7)	0 (0.0)	6 (15.0)	0.03
IIB	9 (12.3)	11 (10.0)	11 (14.7)	4 (13.3)	5 (12.5)	
IIIA	10 (13.7)	22 (20.0)	16 (21.3)	8 (26.7)	12 (30.0)	
IIIB	46 (63.1)	61 (55.5)	42 (56.0)	17 (56.7)	17 (42.5)	
IIIC	0 (0.0)	10 (9.1)	1 (1.3)	1 (3.3)	0 (0.0)	
**Histological variety**						
Ductal	61 (83.5)	98 (89.2)	73 (97.4)	30 (100)	38 (95.0)	0.08
Lobular	7 (9.6)	8 (7.2)	1 (1.3)	0	1 (2.5)	
Other	5 (6.9)	4 (3.6)	1 (1.3)	0	1 (2.5)	
Histological grade^*^						
1	24 (32.8)	9 (8.2)	8 (10.7)	3 (10.0)	2 (5.0)	0.001
2	38 (52.0)	39 (35.5)	26 (34.7)	9 (30.0)	10 (25.0)	
3	11 (15.2)	62 (56.4)	41 (54.7)	18 (60.0)	28 (70)	
**Tumour size (mm)**						
< 30	4 (5.7)	5 (4.8)	7 (10.6)	0 (0.0)	3 (7.9)	0.32
≥ 30	66 (94.3)	99 (95.2)	59 (89.4)	29 (100)	35 (92.1)	
Type of surgery^*^						
Quadrantectomy	22 (30.1)	33 (30.0)	23 (30.7)	4 (13.3)	20 (50.0)	0.02
Mastectomy	51 (69.9)	77 (70.0)	52 (69.3)	26 (86.7)	20 (50.0)	
Sentinel lymph node^*^	3 (4.1)	7 (6.4)	10 (13.3)	1 (3.3)	7 (17.5)	0.04
**Axillary clearance**	70 (95.9)	104 (94.6)	69 (92.0)	30 (100)	34 (85.0)	0.08
Positive lymph nodes at clearance^*^						
Negative	23 (32.9)	33 (31.7)	31 (44.9)	14 (46.7)	19 (55.9)	0.002
1 to 3	25 (35.7)	26 (25.0)	24 (34.8)	9 (30.0)	4(11.8)	
4 to 9	19 (27.1)	23 (22.1)	8 (11.6)	2 (6.7)	8 (23.5)	
≥ 10	3 (4.3)	22 (21.2)	6 (8.7)	5 (16.7)	3 (8.8)	
KI-67 score^*^						
≤14	73 (100)	0 (0.0)	38 (50.7)	14 (46.7)	15 (37.5)	0.001
>14	0 (0.0)	110 (100)	37 (49.3)	16 (53.3)	25 (62.5)	
**HER2/neu–Trastuzumab**						
HER2/neu–Yes Trastuzumab	NA	NA	64 (85.3)	25 (83.3)	NA	0.79
HER2/neu–No Trastuzumab	NA	NA	11 (14.7)	5 (16.7)	NA	
**Neoadjuvant chemotherapy regimen**						
Anthracycline based only	8 (11.0)	18 (16.4)	15 (20.0)	1 (3.3)	4 (10.0)	0.20
Anthracycline and taxane based	65 (89.0)	86 (78.2)	58 (77.3)	29 (96.7)	34 (85.0)	
Taxane-based only	0 (0.0)	3 (2.7)	1 (1.3)	0 (0.0)	2 (5.0)	
CFM^**^	0 (0.0)	3 (2.7)	1 (1.3)	0 (0.0)	0 (0.0)	
**Metastasis and/or relapse**						
Local	1 (12.5)	5 (11.4)	2 (14.3)	0 (0.0)	1 (20.0)	0.89
Metastasis	7 (87.5)	39 (88.6)	12 (85.7)	5 (100)	4 (80.0)	

* <0.05, IQR: interquartile range,

** CFM: cyclophosphamide, methotrexate, and fluorouracil

**Table 4.** Multivariable Cox regression models to overall survival (OS) and disease-free survival (DFS).

Characteristics	OS			DFS		
	HR	95% CI	*p* value	HR	95% CI	*p* value
**Response to NAC**						
Complete response	1.00			1.00		
No response	5.43	1.26–23.3	0.02	3.96	1.21–12.9	0.02
**Intrinsic subtypes**						
Luminal A	1.00			1.00		
Luminal B	5.12	2.18–12.05	0.01	3.19	1.66–6.14	0.01
Luminal B/HER2+	1.58	0.57–4.38	0.37	1.28	0.59–2.76	0.51
HER2 - enriched	2.84	0.91–8.83	0.07	1.60	0.63–4.03	0.31
Triple negative	7.41	2.73–20.14	0.01	2.62	1.14–6.00	0.02
**Histological grade**						
1 and 2		No significant		1.00		
3				1.66	1.09–2.54	0.01
**Clinical stage**						
II	1.00				No significant	
III	3.43	1.38–8.54	0.01			
